# Unilateral adrenalectomy for a drug-resistant bilateral primary aldosteronism with heart failure: pathophysiology and surgical indication

**DOI:** 10.1186/s12902-023-01503-2

**Published:** 2023-11-07

**Authors:** Seiji Hoshi, Akifumi Onagi, Ryo Tanji, Ruriko Honda-Takinami, Kanako Matsuoka, Junya Hata, Yuichi Sato, Hidenori Akaihata, Masao Kataoka, Soichiro Ogawa, Yoshiyuki Kojima

**Affiliations:** Departments of Urology, Fukushima Medical University School of Medicine, Fukushima, Japan

**Keywords:** Primary aldosteronism, Laparoscopic adrenalectomy, Heart Failure

## Abstract

**Background:**

Patients with bilateral primary aldosteronism (PA) generally are treated with antihypertensive drugs, but optimal treatment for patients with complications due to refractory hypertension has not been established. In this report, we present a case with bilateral PA who presented with persistent hypertension, despite treatment with 6 drugs, and left-dominant heart failure, which was improved after unilateral adrenalectomy.

**Case presentation:**

A 61-year-old man was admitted to our hospital because of severe left-dominant heart failure. His heart rhythm was atrial fibrillation and the left ventricle was diffusely hypertrophic and hypokinetic. Coronary arteries were normal on coronary arteriogram. Primary aldosteronism was suspected based on severe hypokalemia (2.5 mEq/L) and plasma aldosterone concentration (PAC; 1,410 pg/mL). Although computed tomography (CT) showed a single left cortical nodule, adrenal vein sampling (AVS) indicated bilateral PA. Early in the case, heart failure and hyperkalemia in this patient were improved by treatment with a combination of 6 antihypertensive drugs (spironolactone 25 mg/day, eplerenone 100 mg/day, azosemide 60 mg/day, tolvaptan 7.5 mg/day, enalapril 5 mg/day, and bisoprolol fumarate 10 mg/day); however, heart failure relapsed after four months of treatment. We hypothesized that hypertension caused by excess aldosterone was inducing the patient’s heart failure. In order to reduce aldosterone secretory tissue, a laparoscopic adrenalectomy was performed for the left adrenal gland, given the higher level of aldosterone from the left gland compared to the right. Following surgery, the patient’s heart failure was successfully controlled despite the persistence of high PAC. Treatment with anti-hypertensive medications was reduced to two drugs (eplerenone 100 mg/day and bisoprolol fumarate 10 mg/day). In order to elucidate the mechanism of drug resistance, immunohistochemistry (IHC) and real time-polymerase chain reaction (RT-PCR) assays were performed to assess the expression of steroidogenic factor 1 (SF-1), a regulator of steroid synthesis in adrenal tissue. IHC and RT-PCR demonstrated that the expression of SF-1 in this patient (at both the protein and mRNA levels) was higher than that observed in unilateral PA cases that showed good responsivity to drug treatment.

**Conclusions:**

Unilateral adrenalectomy to reduce aldosterone secretory tissue may be useful for patients with drug-refractory, bilateral PA. Elevated expression of SF-1 may be involved in drug resistance in PA.

## Introduction

Primary aldosteronism (PA) is one of the most pervasive causes of secondary hypertension [1]. Aldosterone-producing adenoma (APA) and idiopathic hyperaldosteronism are the common subtypes of PA, comprising approximately 65% and 30% of cases, respectively [2]. In general, APA is a unilateral disease; bilateral APA is extremely rare. The overall treatment goal in patients with PA is to prevent the morbidity and mortality associated with hypertension, hypokalemia, renal toxicity, and cardiovascular damage [3]. In general, unilateral disease is considered surgically curable, whereas bilateral disease typically is treated with mineralocorticoid receptor (MR) antagonists [3].

Although aldosterone initially induces sodium and water retention, these effects are followed within a few days by a spontaneous diuresis (called aldosterone escape) that returns excretion to the level of intake and partially restores the extracellular fluid volume to normal levels [4–6]. Therefore, heart failure associated with PA had been considered rare. However, a recent meta-analysis indicated that patients with PA have an increased risk of cardiovascular events and heart failure compared to individuals with essential hypertension [7]. Therefore, it is important to consider appropriate treatment for PA with heart failure.

Bilateral PA patients are rare; such individuals sometimes show refractory hypertension, even when treated with MR antagonists and more than three antihypertensive drugs [1], and optimal treatment of these patients remains unclear. Herein, we present a case with bilateral APA who presented with persistent hypertension, despite treatment with 6 drugs, and exhibited left-dominant heart failure; this individual’s condition improved following unilateral adrenalectomy. In addition, we discuss the mechanism of drug resistance and improvement of persistent hypertension and heart failure after unilateral adrenalectomy in this case.

### Statistical analysis

Determination mRNA expression level and western blotting analysis were repeated at least three times independently, and the results were expressed as the mean ± SE. Analysis was performed with SPSS Statistics 21 software (IBM Japan). Data were statistically evaluated using the unpaired two-tailed Student’s t test and values were considered statistically significant when P < 0.05.

### Case presentation

**Fig. 1:**
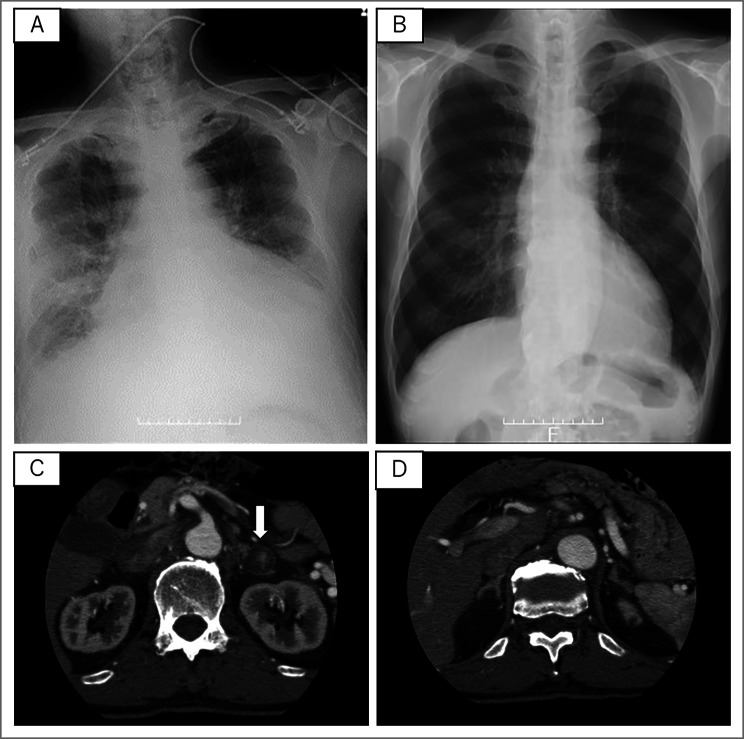
Chest x-ray and adrenal CT images. Preoperative chest x-ray showing pleural effusion and enlarged heart (**A**). Postoperative chest x-ray showing improvement of pleural effusion and of cardiac enlargement (**B**). Computed tomography (CT) showing presence of a 25 mm × 23 mm × 22 mm single adrenal nodule on the left gland (**C**) and absence of adrenal nodule on the right gland (**D**)

A 61-year-old man was admitted to our hospital because of severe left-dominant heart failure. Chest radiography showed an enlarged cardiac silhouette, a congested pulmonary hilum, and bilateral pleural effusion (Fig. 1A). This patient’s heart rhythm was atrial fibrillation, and the left ventricle was diffusely hypertrophic and hypokinetic. Coronary arteries were normal on coronary arteriogram. Heart failure was improved by administration of cardiotonic and diuretic drugs, and PA was suspected given the existence of severe hypokalemia (2.5 mEq/L), suppression of plasma renin activity (< 0.1 ng/ml/h) and elevated plasma aldosterone concentration (PAC, 1,410 pg/mL) (Table 1). Computed tomography (CT) revealed the presence of a single left adrenal nodule (25 mm × 23 mm × 22 mm; Fig. 1C), but failed to detect any adrenal nodule on the right gland (Fig. 1D). However, adrenal vein sampling (AVS) indicated bilateral PA (Table 2); therefore, treatment with medication was begun. Initially, heart failure and serum potassium levels (4.2 mEq/L) were improved by administration of the combination of two MR antagonists (spironolactone 25 mg/day and eplerenone 100 mg/day), two diuretics (azosemide 60 mg/day and tolvaptan 7.5 mg/day), an angiotensin-converting-enzyme inhibitor (enalapril 5 mg/day), and a beta-adrenergic blocker (bisoprolol fumarate 10 mg/day). However, heart failure relapsed after 4 months of treatment. We hypothesized that excess aldosterone might be the source of hypertension and heart failure in this patient. In order to reduce the amount of aldosterone secretory tissue, a laparoscopic adrenalectomy was performed for the left adrenal gland, which was observed to secrete more aldosterone than the right gland (6,950 and 2,130 pg/dL, respectively; Table 2). This procedure was performed with an operation time of 2 h and 5 min and a bleeding volume of 20 mL. The resected adrenal tissue consisted of an encapsulated yellowish nodule. Histopathological examination revealed a benign adrenal adenoma without hyperplasia in the normal adrenal region, which was diagnosed as an aldosterone-producing adenoma.

**Table 1:** Laboratory characteristics

	Upon admission	Recovering from heart failure (after 2 months)	Before surgery (after 7 months)	After surgery (after 8 months)	Reference values
WBC (10^3/µL)	5.1	4.0	7.1	4.1	3.8–9.8
RBC (10^6/µL)	4.09	3.46	3.55	3.55	4.16–5.58
Hb (g/dL)	11.4	9.6	10.4	11.3	13.2–16.8
BUN (mg/dL)	18.7	34	36	35	8–22
Cre (mg/dL)	1.05	1.61	1.70	1.45	0.60–1.10
Na (mmol/L)	144	137	140	135	138–146
K (mmol/L)	2.5	4.2	3.3	4.9	3.6–4.9
Cl (mmol/L)	107	101	103	102	99–109
ACTH (pg/mL)	17.6	28.6	29.4	42.02	
PRA (ng/ml/h)	< 0.1	< 0.1	< 0.1	0.3	
PAC (pg/mL)	661	523	1410	293	
ARR	6610	5230	14,100	976.6	
Cortisol (µg/dL)	10.8	13.6	12.8	13.21	
DHEA (µg/dL)	15	39	37	52	
BNP (pg/mL)	2254.4	231.0	4919.0	104.4	0-18.4

WBC: white blood cell count, RBC: red blood cell count, Hb: hemoglobin, BUN: blood urine nitrogen

Cre: creatinine, ACTH: adrenocorticotropic hormone, PRA: plasma renin activity, PAC: plasma aldosterone concentration, ARR: aldosterone-to-renin ratio, DHEA: dehydroepiandrosterone, BNP: brain natriuretic peptide

**Table 2:** Results of adrenal venous sampling

Pre-ACTH Loading			
	Aldosterone (pg/dL)	Cortisol (nmol/L)	A/C
LAV	6,950	23.3	298.3
RAV	2,130	18.6	114.5
IVC	496	9.3	53.3
LAV: RAV ratio (left/ right)	2.1	1.25	
**Post-ACTH Loading**			
	**Aldosterone** **(pg/dL)**	**Cortisol** **(nmol/L)**	**A/C**
LAV	53,400	1540	34.7
RAV	34,700	1000	34.7
IVC	692	25.9	26.7
LAV: RAV ratio (left/ right)	1.5	1.54	

Result of adrenal venous sampling. This result indicates aldosterone hypersecretion from bilateral adrenal gland and its secretion large in left. LAV: left adrenal vein, RAV: right adrenal vein, IVC: inferior vena cava, A/C: PAC/Cortisol ratio

**Fig. 2:**
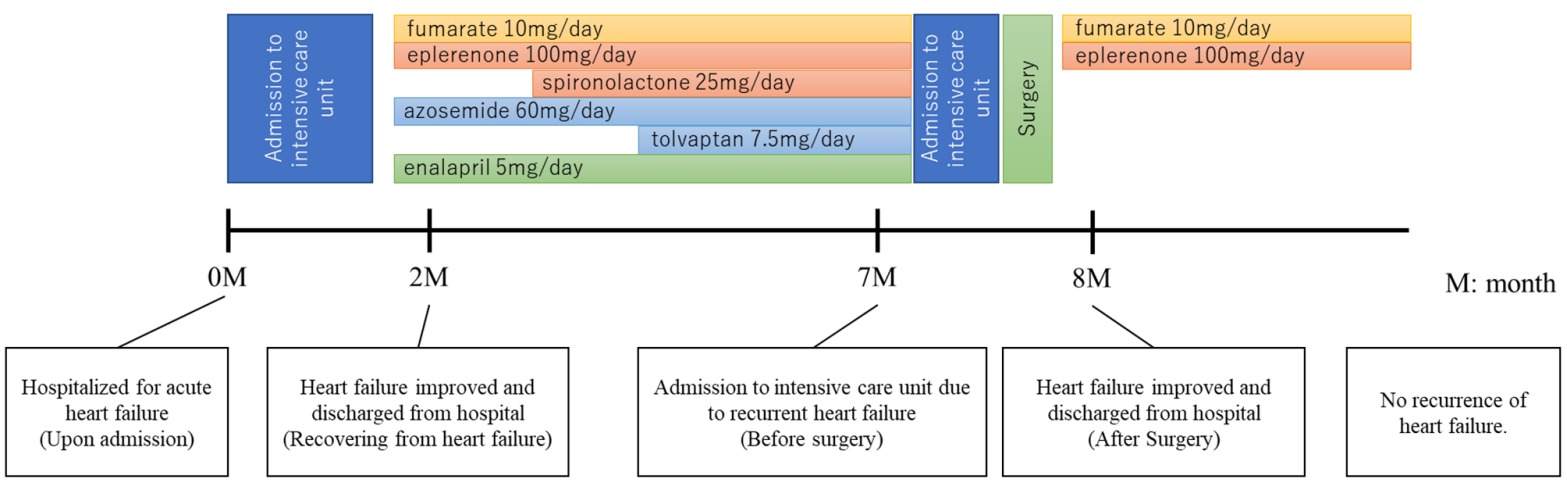
Summary of the treatment in this case. A summary of this case’s progress from initial admission to the present

Immediately after surgery, the patient’s heart failure was successfully controlled, despite the fact that the PAC remained elevated (293 pg/mL, Table 1). The number of antihypertensive drugs was reduced to two, consisting of eplerenone 100 mg/day and bisoprolol fumarate 10 mg/day. Chest x-ray showed improvement of both pleural effusion and cardiac enlargement (Fig. 1B), and postoperative cardiac ultrasound demonstrated improvement of ventricular hypertrophy (preoperative left ventricular end-diastolic/systolic diameter (LVDd/Ds) 69.5/49.6 mm, postoperative LVDd/Ds 57.3/45.7 mm). Two years after surgery, although serum PRA was low (0.3 ng/ml/h) and PAC was high (249 pg/mL) and CT showed scattered small 1–2 mm hypo-absorptive lesions suggestive of microadenoma in the contralateral adrenal gland, heart failure has not relapsed. A summary of the treatment of this patient is provided in Fig. 2.

**Fig. 3:**
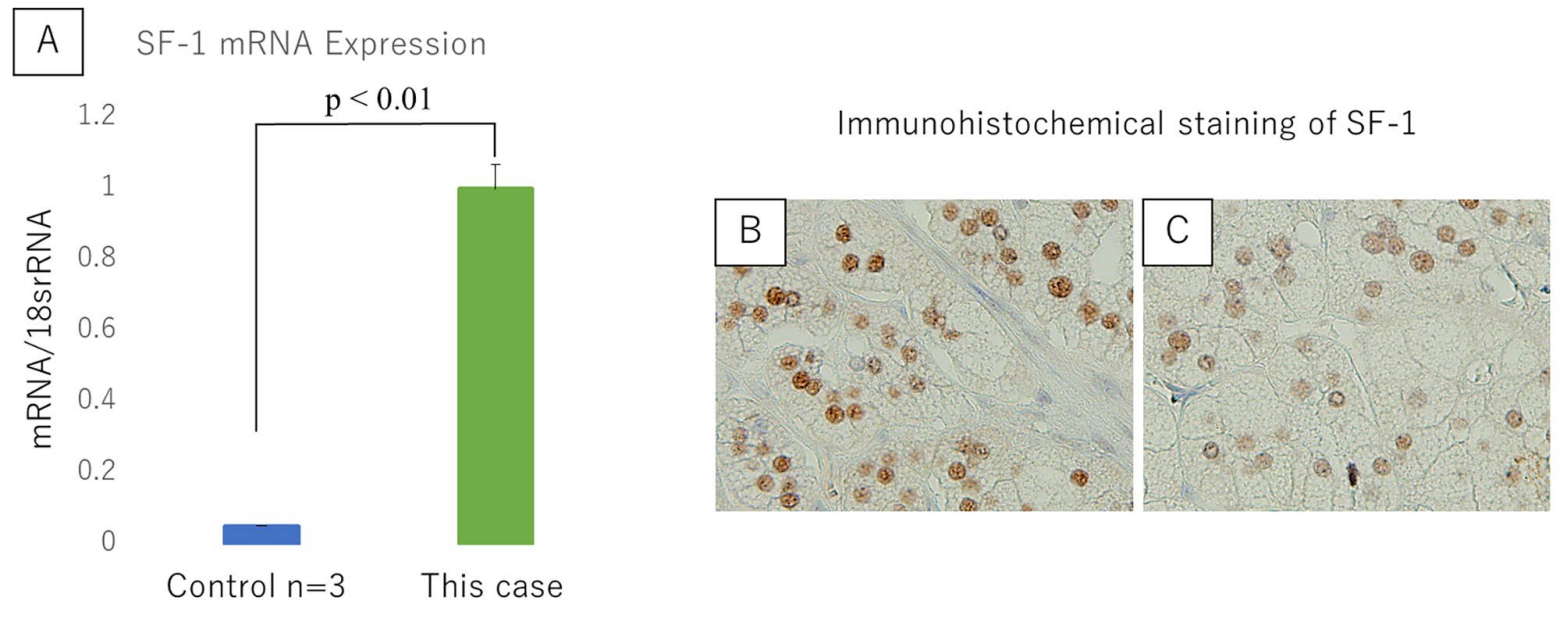
Expression analysis of SF-1. Expression analysis of *SF-1* mRNA by RT-PCR (**A**) and of SF-1 protein by IHC (**B** and **C**) in controls (tissue from patients with aldosterone-producing adenoma, n = 3) and in tissue from the subject of the present case report. *SF-1* mRNA levels were significantly higher in the present case than in the controls (**A**). Statistical analysis was performed with the Mann-Whitney U test. In IHC, nuclear staining was nominally higher in the present case (**B**) than in the controls (**C**). IHC: immunohistochemical staining, RT-PCR: reverse transcription-polymerase chain reaction, SF-1: steroidogenic factor 1

To elucidate the mechanism of drug resistance, quantitative reverse transcription-polymerase chain reaction (RT-PCR) and immunohistochemistry (IHC) assays were performed on the resected tissue to detect the mRNA and protein expression (respectively) of steroidogenic factor 1 (SF-1), a regulator of steroid synthesis in adrenal tissue (Fig. 3). Tissue from unilateral PA cases with good responsivity to drug treatment were used as controls (n = 3). The preoperative PRA, PAC, and tumor length in the three control patients were,<0.1,<0.1,<0.1 ng/ml/h, 568, 670, 714 pg/mL, and 14, 25, 28 mm, respectively. RT-PCR (Fig. 3A) and IHC (Fig. 3B and C) showed that SF-1 mRNA and protein expression levels were significantly higher in this case than those in the controls (p < 0.01 by Mann-Whitney U test).

## Discussion and conclusions

Primary aldosteronism is a disease that causes hypertension and hypokalemia; generally, PA can be controlled with anti-aldosterone drugs such as MR antagonists [3]. However, there have been reports of heart failure in some PA patients with uncontrolled hypertension. In such cases, there is no therapeutic approach aside from surgical treatment, though few reports have shown that heart failure was improved by performing unilateral adrenalectomy in patients with unilateral PA [1, 8]. To the best of our knowledge, the case described here is the first case in which heart failure was improved by performing unilateral adrenalectomy for bilateral APA.

Bilateral surgical removal generally is not recommended for bilateral PA [3]. This caution reflects the fact that bilateral removal of adrenal glands necessitates lifelong steroid hormone replacement therapy following the surgery. On the other hand, there appear to have been few reports on the effectiveness of mass-reduction surgery for the treatment of bilateral PA, a strategy that was employed in the case described in the present report. Norlela et al. reported the effectiveness of unilateral resection for 40 cases of bilateral PA [9]. That work indicated that a significant reduction in PAC and normalization of blood pressure was achieved in 65% of patients, but only 15% of patients were able to normalize their blood pressure without the subsequent need for drugs. Those results suggested that unilateral adrenalectomy for patients with bilateral PA may reduce the total secretion of aldosterone, facilitating the subsequent normalization of blood pressure in most cases. In the present case, we conjectured that hypertension was due to excessive secretion of aldosterone, which adversely affected aortic regurgitation and induced heart failure. The observed improvement of heart failure presumably reflected a decrease in the total secretion of aldosterone and of blood pressure following unilateral adrenalectomy. Thus, if hypertensive complications are uncontrolled in patients with bilateral PA, unilateral adrenalectomy should be considered as a potential treatment option. Finally, the diagnosis of bilateral disease is difficult, and when the AVS results deviate from the imaging findings, it is necessary to consider reconstructing the AVS. In the present case, retesting of AVS was also considered, but surgical treatment was preferred due to the patient’s condition. Based on postoperative imaging and PAC, this patient was considered to have APA on the left and microadenoma on the right.

In the case described here, 6 antihypertensive drugs were needed to control refractory hypertension before surgery. There are individual differences in the efficacy of antihypertensive drugs such as anti-aldosterone drugs in PA, and the actual mechanism of action of these medications remains poorly understood. SF-1 is a regulator of steroid synthesis, and overexpression of SF-1 in adrenal tissue has been shown to promote steroid hormone biosynthesis [10–12]. Several reports from in vivo studies have indicated that elevation of SF-1 may play an important role in APA formation and PA [13, 14]. Notably, our analysis showed that SF-1 was expressed at higher levels in the patient described here than in control cases. We hypothesize that elevated expression of SF-1 induces unregulated aldosterone secretion by promoting steroid hormone biosynthesis, resulting in drug-resistant refractory hypertension.

## Data Availability

Data sharing is not applicable to this article as no datasets were generated or analyzed during the current study.

## Abbreviations

APA: Aldosterone-producing adenoma
AVS: Adrenal vein sampling
IHC: Immunohistochemistry
LVDd/Ds: Left ventricular end-diastolic/systolic diameter
MR: Mineral corticoid receptor
PA: Primary aldosteronism
PAC: Plasma aldosterone concentration
RT-PCR: Real time-polymerase chain reaction
SF-1: Steroidogenic factor 1
